# Hospital Meetings

**Published:** 1900-11-24

**Authors:** 


### HOSPITAL MEETINGS.

BRITISH HOME FOR INCURABLES.

A half-yearly meeting of the British Home and Hospital for Incurables was held at the Institution, Streatham Common, on Thursday, 15th inst., Colonel Clifford Gascoyne presiding. The chief business to be transacted was the announcement of the result of the recent election, at which five inmates and eleven pensioners were selected; and the secretary having read the names of these, the chairman remarked that since the last meeting the new wing had been completed and opened, and they were now able to accommodate eighty persons—considerably more than ever before. The customary votes of thanks brought the proceedings to a close.

